# Comparison of the appendicitis inflammatory response and Alvarado scoring systems in the diagnosis of acute appendicitis in children

**DOI:** 10.25122/jml-2020-0031

**Published:** 2021

**Authors:** Mohammad Vaziri, Nahid Nafissi, Fariba Jahangiri, Mohammad Nasiri

**Affiliations:** 1. Department of Surgery, Hazrat-e Rasoul Akram Hospital, Iran University of Medical Sciences, Tehran, Iran; 2. Department of Breast Surgery, Hazrat-e Rasoul Akram Hospital, Iran University of Medical Sciences, Tehran, Iran; 3. Department of Pediatric Surgery, Ali-Asghar children Hospital, Iran University of Medical Sciences, Tehran, Iran

**Keywords:** appendicitis, Alvarado, AIR, pediatric

## Abstract

Our objective was to compare the diagnostic accuracy of Alvarado and appendicitis inflammatory response (AIR) scoring systems among children suspected of acute appendicitis concerning their postoperative outcomes. During a two-year period, a prospective multicentric study was carried in the selected hospitals of Iran. All children who were admitted with the diagnosis of acute appendicitis were enrolled in the study. However, patients suffering from generalized peritonitis or those who had a history of abdominal surgery were excluded. Before decision-making, each patient’s score according to two appendicitis scoring systems was calculated. The clinical outcomes and diagnosis of patients were then compared to the results of each scoring system. For those patients who were a candidate for surgery, the final diagnosis of acute appendicitis was made by histopathology. Patients were divided into a high- and low-risk group according to scoring systems outcomes. Among the patients with a low score for appendicitis, the AIR scoring system had a sensitivity and specificity of 95% and 74%, respectively, which was more promising in comparison to that of the Alvarado system (90% and 70%, respectively). Regarding the patients at higher risk of acute appendicitis, none of the scoring systems provided reliable results since both systems showed sensitivity and specificity of less than 50%, which was not sufficient to distinguish patients who are a candidate for surgery. AIR and Alvarado scoring systems are not accurate models to predict the risk of acute appendicitis among children; however, the AIR system could be used as a reliable material to rule out the acute appendicitis diagnosis.

## Introduction

Acute appendicitis is the most common diagnosed cause of acute abdominal pain requiring an urgent surgical intervention urgent to remove the appendix, with an estimated lifetime incidence ranging from 7% to 9% [1]. To avoid severe and progressive inflammation as well as subsequent perforation of the appendix, surgical resection of the appendix has been the treatment of choice for more than a century [2, 3]. However, despite many advances that have been made to improve diagnostic accuracy, the vague and atypical onset of signs and symptoms of appendicitis prohibit the early diagnosis and intervention [4, 5]. 

On the other hand, one-third of children suffering from acute appendicitis do not present typical clinical manifestations such as abdominal pain, nausea and vomiting [6, 7]. Thus, the early and accurate diagnosis might be of great importance among younger patients, who have been recently suggested to receive non-operative treatments rather than surgical interventions, since recent studies do not consider appendicitis to be an irreversible, progressive disease [8, 9]. However, the life-threatening nature of the appendicitis complications, such as perforation, phlegmon formation, and peritonitis, has resulted in an increased number of negative appendectomies, considering its lower morbidity and mortality rate compared to complications [10]. On this basis, a meticulous diagnostic tool is required in pediatric patients with appendicitis to accurately differentiate the patients who require surgical treatment from the patients who could be managed conservatively [11, 12]. Several scoring systems and models have been suggested to play a role in improving the diagnostic accuracy among patients with acute appendicitis, including the Alvarado, Lintula, Fenyo-Lindberg, and RIPASA scoring systems [13, 14]. Although these scoring systems and algorithms have been introduced to the classification of the patients with appendicitis as the main diagnosis according to clinical and paraclinical findings, utilizing them among pediatric patients remains challenging due to the diversity of the clinical manifestations [15, 16]. In addition, scarce data on the pediatric population has prevented the development of diagnostic criteria and models for treating small patients with appendicitis. 

To our knowledge, few studies have evaluated the role of scoring systems in discrimination of treatment approach among pediatric patients with acute appendicitis. The present study aimed to compare the accuracy of Alvarado and AIR scoring systems for the diagnosis of appendicitis, with due attention to the postoperative outcomes.

## Material and Methods

The present prospective multicentric study was carried out between 2017 and 2019 in the selected hospitals of the Iran provinces at the Shahid Beheshti University of Medical Sciences, Tehran, Iran. All children who were admitted with the diagnosis of acute appendicitis were enrolled in the study. However, patients suffering from generalized peritonitis, and those who had undergone previous intra-abdominal surgery, were excluded. 

All demographic and clinical information of the patients were collected using the designated questionnaire, including abdominal pain features, intensity, pain relocation or migration, nausea and vomiting, anorexia, body temperature and fever, tenderness, rebound tenderness, guarding, bowel sounds, total white blood cell counts and differentials, and histopathology outcomes of the patients, who underwent surgery. Prior to patients’ examination, attending physician or surgery residents, who were trained on the two appendicitis scoring systems of Alvarado and AIR as well as the cut-off points for diagnosis, calculated patients’ risk of appendicitis [17, 18]. The diagnostic and evaluation criteria of the two scoring systems are listed in Table 1.

**Table 1:** Scoring systems criteria and cut-off points.

			**Alvarado**	**AIR**
**Symptoms**		**Nausea/Vomiting**	1	
		**Nausea**		1
		**Anorexia**	1	
		**Migration of pain to RLQ**	1	
**Signs**	**Pain in RLQ**		2	1
	**Rebound tenderness**		1	
		**Mild**		1
		**Moderate**		2
		**Severe**		3
	**BT>37.5°C**		1	
	**BT>38.5°C**			1
**Laboratory tests**	**Leukocytosis shift**		1	
	**PMN Leukocytosis**	**70–84%**		1
		**>85%**		2
	**WBC count**	**>10 x 109**	2	
		**10–14.9 x 109**		1
		**>15 x 109**		2
	**CRP concentration**	**10–49 g/L**		1
		**>50 g/L**		2
**Risk of Appendicitis**		**Low risk**	1–4	0–4
		**Intermediate risk**	5–6	5–8
		**High risk**	7–10	9–12

AIR – Appendicitis Inflammatory Response; RLQ – Right Lower Quadrant; BT – Body Temperature; PMN – Polymorphonuclear; WBC – White Blood Cell; CRP – C-Reactive Protein.

During diagnostic workups, all patients underwent abdominal ultrasonography in order to determine the diagnosis; however, in case of unclear outcomes, abdominal computed tomography was indicated for meticulous evaluation of the appendix and inflammation process. For all the patients admitted to the emergency room, the decision-making process was finalized by the attending physician, including discharge, observation, diagnostic tests and paraclinical, or surgical management. The clinical outcomes and diagnosis of patients were then compared to the results of each scoring system. For those patients who were a candidate for surgery, the final diagnosis of acute appendicitis was made by histopathology. Patients who were adjudged not to have appendicitis by the attending clinician were discharged and prescribed analgesics. 

### Statistical analysis

To evaluate the diagnostic accuracy of scoring systems compared to postoperative diagnosis, the sensitivity, specificity, positive predictive value (PPV), and negative predictive value (NPV) were calculated. The disease prevalence was considered to be 0.1% in the general population, according to the literature [19]. Receiver Operating Characteristics (ROC) curve analysis was carried out. The significance level was set at 0.05, and all results were expressed by frequency (percent) for qualitative variables and Mean±SD (standard deviation) for quantitative variables. All analyses were carried out using the SPSS software, version 25.

## Results

**Figure 1.**
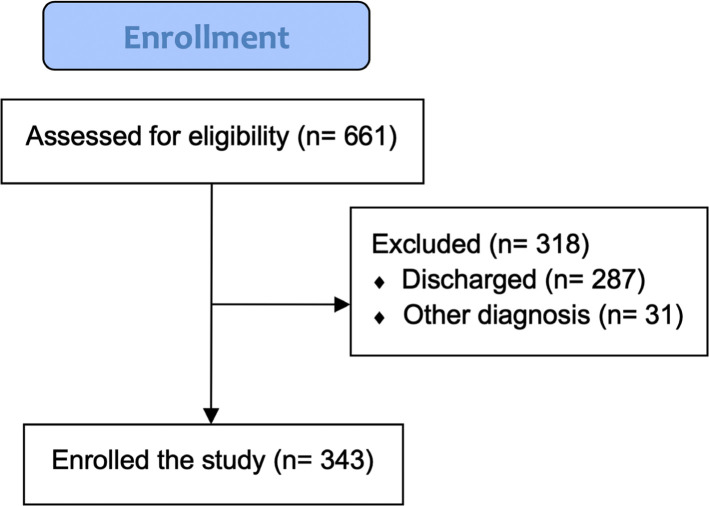
Flowchart of the patients enrolled in the study.

In the current study, 661 children patients who were clinically evaluated for cute abdominal pain were enrolled. In total, 265 boys (40%) and 396 girls (60%) were admitted to the emergency room to confirm or rule out the diagnosis of acute abdomen, with a mean age of 8.9 years (SD= 3.44). The youngest patient was 11 months old, and the oldest patient was 18 years old. After assessing clinical signs and symptoms, a total of 343 (51.8%) children Awere underwent surgery (Figure 1). Subsequently, none of the acute appendicitis cases were missed during clinical evaluation at the emergency room. However, intraoperative observations revealed negative appendectomy in 31 (9%) patients. Of the patients who were in need of an acute laparoscopic or open laparotomy, acute appendicitis was detected in 218 (69.8%) patients. Phlegmonous appendicitis developed in 74 (30.2%) patients. Also, diagnostic imaging was performed in 389 cases (52%). The most frequent alternative diagnosis was nonspecific abdominal pain in 184 (57.8%) patients, followed by gastroenteritis in 97 (30.5%) patients, chronic constipation in 26 (8.1%) patients, and intussusception in 11 (3.4%) patients.

Since each scoring system benefits a different cut-off point to distinguish the risk of appendicitis, the analysis was performed in two distinct subgroups for each scoring system, including patients with a lower and higher risk of acute appendicitis. In Table 2, the sensitivities, specificities, PPVs, and NPVs for scoring systems diagnostic accuracy in different subgroups in predicting appendicitis probability have been shown. Among the patients with scores that determined a lower appendicitis risk, the analysis revealed the AIR scoring system as the best performing model in the prediction of acute appendicitis, with an accuracy of 74.27% (CI95%= 70.75% to 77.56%). However, the area under the curve (AUC) showed a slightly lower accuracy rate for Alvarado, which was 70% (CI 95%= 66.53% to 73.64%) compared to the AIR scoring system.

**Table 2:** Diagnostic accuracy of the scoring systems based on the patients’ risk of developing appendicitis.

		**Sensitivity**	**Specificity**	**Positive Likelihood Ratio (PLR)**	**Negative Likelihood Ratio (NLR)**	**Positive Predictive Value (PPV)**	**Negative Predictive Value (NPV)**	**Accuracy**
**Alvarado**	**High**	43% (CI95%: 37.48–48.58)	32% (CI95%: 26.98–37.51)	0.64 (CI95%: 0.55–0.73)	1.77 (CI95%: 1.47–2.13)	0.16% (CI95%: 0.14–0.18)	99.56% (CI95%: 99.47–99.63)	32%
	**Low**	90% (CI95%: 86.42–93.04)	70% (CI95%: 64.77–75.11)	3.02 (CI95%: 2.54–3.58)	0.14 (CI95%: 0.10–0.20)	0.75% (CI95%: 0.63–0.89)	99.96% (CI95%: 99.95–99.97)	70%
**AIR**	**High**	48% (CI95%: 42.13–52.95)	25% (CI95%: 20.19–29.97)	0.63 (CI95%: 0.56–0.72)	2.11 (CI95%: 1.70–2.62)	0.16% (CI95%: 0.14–0.18)	99.47% (CI95%: 99.35–99.58)	25%
	**Low**	95% (CI95%: 91.83–96.86)	74% (CI95%: 69.04–78.93)	3.67 (CI95%: 3.04–4.44)	0.07 (CI95%: 0.04–0.11)	0.91% CI95%: 0.76–1.10)	99.98% (CI95%: 99.97–99.99)	74%

AIR – Appendicitis Inflammatory Response.

Considering the patients with a high risk of developing acute appendicitis, none of the scoring systems provided an acceptable specificity rate. Despite an extremely high NPV, both scoring models had an interestingly low PPV. An AIR score of 7 or greater showed a sensitivity and specificity of 43% and 32%, respectively, which was comparable to that of the Alvarado system, which was 48% and 25%, respectively. However, neither the Alvarado – accuracy: 32% (CI95%= 28.56% to 35.81%) nor the AIR – accuracy: 25% (CI95%= 21.65% to 28.38%) scoring models could outperform the other group in predicting appendicitis in pediatric patients who had a score of 7 points or greater in both groups.

## Discussion

Since the introduction of clinical scoring systems of acute appendicitis, they have played a crucial role as a predictive tool in the decision-making process in patients suspected of appendicitis to estimate the probability of the disease [20, 21]. The simple design, feasibility, and applicability of the scoring systems contribute to rapid decision-making by providing a suitable instrument for patients’ selection to carry out further diagnostic tests, including laboratory and imaging workups [22]. Although several studies have evaluated and compared the accuracy of scoring systems and models in adult patients suffering from appendicitis, few studies have assessed the scoring systems to determine the most reliable tool among children [23, 24]. In addition, considering their clinical signs and symptoms, the classification might be challenging among children with due attention to atypical and vague manifestations and lack of sufficient accuracy among imaging tools, such as ultrasonography [25, 26]. Furthermore, despite recent advances in the diagnosis of appendicitis after enrolling the computed tomography (CT) scan in the diagnosis process, preventing the vast exposure to ionizing radiation in children prohibited physicians and clinicians from taking advantage of the imaging methods [27–29]. Therefore, it has been believed that the clinical judgment of a senior surgeon might be superior to the efficacy of scoring systems in distinguishing children who are suspected of appendicitis [30, 31]. Still, there is a demand to compare the different scoring tools to facilitate the approaching process among younger patients [32].

To our knowledge, this is one of the first studies which have prospectively compared the accuracy of the AIR and Alvarado scores in the estimation of appendicitis development in pediatric patients [33, 34]. According to the study results, both of the scoring systems evaluated in the current survey provide acceptable diagnostic accuracy in ruling out the probability of appendicitis among young individuals due to their higher sensitivity and specificity followed by an acceptable discriminative value. However, the accuracy of the AIR scoring system in the meticulous assessment of appendicitis risk was considerably higher in comparison to that of Alvarado scoring. The main differences between the application of the AIR and Alvarado score in pediatric patients are derived from the characteristics of the scoring criteria in the aforementioned scoring systems. Contrary to the Alvarado system, the AIR scoring system focuses on clinical signs and paraclinical outcomes rather than the subjective evaluation of patients’ complaints when selecting those at high probability for acute appendicitis. A high AIR score has excellent specificity and positive predictive values that exceed those of the Alvarado score.

Despite the application of the scoring systems in adult patients suffering from acute appendicitis, which results in better discrimination of the patients at higher risk of appendicitis, both applied scoring systems have provided better outcomes in ruling out the patients with a lower risk of appendicitis between pediatric participants. However, considering the prospective design of our survey, the scoring systems did not play the main role during decision-making for patients’ management and senior surgeon discretion based on clinical manifestations. The Alvarado scoring system revealed a sensitivity and specificity of 90% and 70%, respectively. However, the sensitivity was lower in comparison to that found by a meta-analysis, but we found a noteworthy higher specificity [35]. Besides, our analysis showed that patients with a lower risk of appendicitis would be more accurately stratified by utilizing the AIR scoring system with a sensitivity of 95% and specificity of 74%, which did not satisfy the condition considering the patients at a higher risk of appendicitis with a sensitivity and specificity of 48% and 25%, respectively. In the literature, it has been suggested that the AIR score identifies more confidently those patients with a high probability of appendicitis, which was not consistent with the latest findings in pediatric patients with high suspicion of acute appendicitis [36, 37]. 

Similarly, in an earlier study conducted by Macco *et al.*, the authors reported that AIS had the highest discriminating power in ruling out the patients with a lower risk of acute appendicitis in children [33]. Thus, it can be hypothesized that differential diagnosis of pediatric appendicitis should still be confirmed by an integrative assessment of clinical and imaging findings. However, with due attention to our results, we failed to report an extreme superiority for the AIR scoring system discriminating power in pediatric patients compared to that of the Alvarado system. Although our findings were consistent with a previous study by Musbahi *et al.*, our results were not consistent with the results of Macco *et al.* [33, 34]. Also, they revealed a 14% rate of missed appendicitis, which is not only two-fold higher in comparison to the missed cases evaluated by the Alvarado score but also increases the risk of development of life-threatening complications of appendicitis in children. Therefore, we believe that the application of predictive tools to improve clinical management in children with appendicitis is capable of reducing unnecessary workups, diagnostic procedures, hospitalization, and surgical interventions. However, the best scoring system should be chosen concerning the clinical manifestation of patients and results of paraclinical examinations.

The multicentric design of the study, which included several hospitals from various provinces of the country, could be considered as the main strength of the current survey, followed by a large number of cases, as well as considering the intraoperative findings as the gold standard for the diagnosis. However, our study had some weaknesses, as follows: first, the definition of the symptoms and their onset characteristics might be difficult in children. Second, evaluation of pain intensity and severity of guarding is based on the physician’s judgment, which might be uncertain, particularly in pediatric wards. Third, due to the prospective design of the investigation, we were unable to evaluate the missed appendicitis in the current study since all patients were followed up till their complete recovery.

## Conclusion

Although the AIR and Alvarado scoring systems for distinguishing patients with acute appendicitis cannot be considered an accurate diagnostic material among pediatric patients, our results showed higher accuracy for the AIR scoring system in the discrimination of patients with a lower risk of acute appendicitis. Therefore, a specific pediatric scoring system and criteria are needed to rule out appendicitis in children suspected of acute appendicitis meticulously.

## Acknowledgments

### Ethical approval

The approval for this study was obtained from the Ethics Committee of the Iran University of Medical Sciences (Approval ID: IR.sbmu.970215.17)

### Consent to participate

Informed written consent was collected from the parents of patients.

### Conflict of interest

The authors declare that there is no conflict of interest.
